# Emerging mass spectrometry-based proteomics methodologies for novel biomedical applications

**DOI:** 10.1042/BST20191091

**Published:** 2020-10-20

**Authors:** Lindsay K. Pino, Jacob Rose, Amy O'Broin, Samah Shah, Birgit Schilling

**Affiliations:** 1 Department of Biochemistry and Biophysics, University of Pennsylvania, Philadelphia, PA, U.S.A.; 2 Buck Institute for Research on Aging, Novato, CA, U.S.A.

**Keywords:** mass spectrometry, proteomics, technology

## Abstract

Research into the basic biology of human health and disease, as well as translational human research and clinical applications, all benefit from the growing accessibility and versatility of mass spectrometry (MS)-based proteomics. Although once limited in throughput and sensitivity, proteomic studies have quickly grown in scope and scale over the last decade due to significant advances in instrumentation, computational approaches, and bio-sample preparation. Here, we review these latest developments in MS and highlight how these techniques are used to study the mechanisms, diagnosis, and treatment of human diseases. We first describe recent groundbreaking technological advancements for MS-based proteomics, including novel data acquisition techniques and protein quantification approaches. Next, we describe innovations that enable the unprecedented depth of coverage in protein signaling and spatiotemporal protein distributions, including studies of post-translational modifications, protein turnover, and single-cell proteomics. Finally, we explore new workflows to investigate protein complexes and structures, and we present new approaches for protein–protein interaction studies and intact protein or top-down MS. While these approaches are only recently incipient, we anticipate that their use in biomedical MS proteomics research will offer actionable discoveries for the improvement of human health.

## Introduction

**Figure 1.**
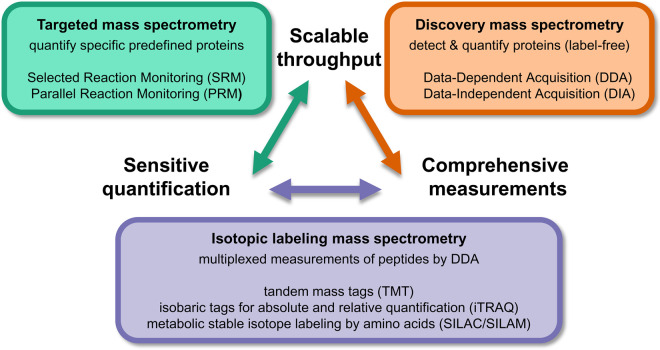
Comparison of MS acquisition methods for proteomics. Protein samples can be analyzed by mass spectrometry using conceptually different methodologies which each require their own unique experimental design. These workflows feature differences in the scalability of sample throughput (the number of samples in a given experiment), in the comprehensiveness of protein and peptide measurements (the number of proteins/peptides that can be detected and quantified), and finally the sensitivity of quantification (the lowest amount of protein/peptide that the method can reliably measure). Targeted mass spectrometry workflows include SRM and PRM (green box) and can measure many samples using optimized assays with deep quantitative sensitivity and accuracy. Label-free discovery mass spectrometry workflows (orange box), such as DDA and DIA may feature overall lower sensitivity for quantification, however, allow to measure proteins more comprehensively in an unbiased approach. DDA workflows are often augmented by sample preparation methods referred to as ‘isotopic labeling’ which feature good quantitative sensitivity and preserve comprehensive acquisitions but are challenged by the maximum number of samples easily achievable in a quantitative experiment.

Developments in both mass spectrometry (MS) hardware and software within the last decade are opening new avenues for the quantitative investigation of proteins involved in human health and disease. Both absolute and relative quantitative measurements, reviewed in detail elsewhere [1], are possible using commonly applied MS acquisition methods. The choice of MS acquisition method influences selectivity, reproducibility, repeatability, limit of detection, dynamic range, and data density [2]. Additionally, the variety of acquisition types places specific requirements on experimental design and strongly influences the computational strategy for analyzing data. A comparison of these different workflows is presented in Figure 1 featuring some of their unique strengths as discussed in detail below.

Generally, MS acquisitions fall into two categories defined by how the mass spectrometer acquires precursor ion scans (MS1) and fragment ion scans (MS/MS or MS2), resulting in either workflows that require no prior knowledge about the proteins in the sample (discovery-based proteomics, sometimes referred to as ‘shotgun’ proteomics) or workflows that rely on a hypothesis to guide the acquisition (targeted proteomics). The discovery acquisition workflow, also referred to as ‘data-dependent acquisition’ (DDA), is defined by how the instrument selects the top N most abundant precursor ions (MS1) for fragmentation and MS/MS analysis during each scan cycle. It typically acquires just one MS/MS spectrum for each precursor ion, as precursor ions are usually dynamically excluded after selection for MS/MS. This results in an analyte-dependent ‘sampling’ and analysis of the peptides in a proteome. These DDA protein discovery experiments can also quantify proteins, when taking advantage of the quantitative MS1-scan signal by extracting the MS1 precursor ion chromatograms that some studies refer to as MS1 filtering [3] or label-free quantification [4]. Alternatively, DDA workflows are often combined with stable isotope labeling techniques to add a quantitative dimension. For example, stable isotope labeling with amino acids in cell culture (SILAC) incorporates heavy isotope lysine and arginine amino acids during cell culture [5]. Isobaric tagging methods, such as tandem mass tagging (TMT) [6,7] (discussed in more detail below) and isobaric tag for relative and absolute quantitation (iTRAQ) [8], are popular for their ease of implementation and amenity to multiplexing samples. Although DDA methods are straightforward to implement and a wealth of analytic software is available for data processing, the stochastic sampling characteristic of DDA often fails to trigger MS/MS spectra acquisition reproducibly from sample to sample, even comparing technical replicate acquisitions, posing challenges for quantification and missing some analytes entirely. On the other hand, several hypothesis-driven, targeted acquisition methods are available. Selected reaction monitoring (SRM) [9], also known as multiple reaction monitoring (MRM) [10], is performed on triple quadrupole-type mass spectrometers. More recently, parallel reaction monitoring (PRM) [11–13] involves high-resolution instruments, such as quadrupole orbitrap MS or quadrupole time of flight (QTOF) instruments. SRM/MRM and PRM methods acquire peptides based on a pre-programmed list of analytes that subsequently allow the use of fragment ions (MS2) for accurate quantification and often results in higher specificity and selectivity. A large variety of informatics tools have been developed to aid in assay development and to process the data collected by various acquisition types [14,15]. Novel bioinformatics tools include quality control algorithms [16], *de novo* peptide sequencing [17], and web-based resources for dissemination of results [18].

**Figure 2.**
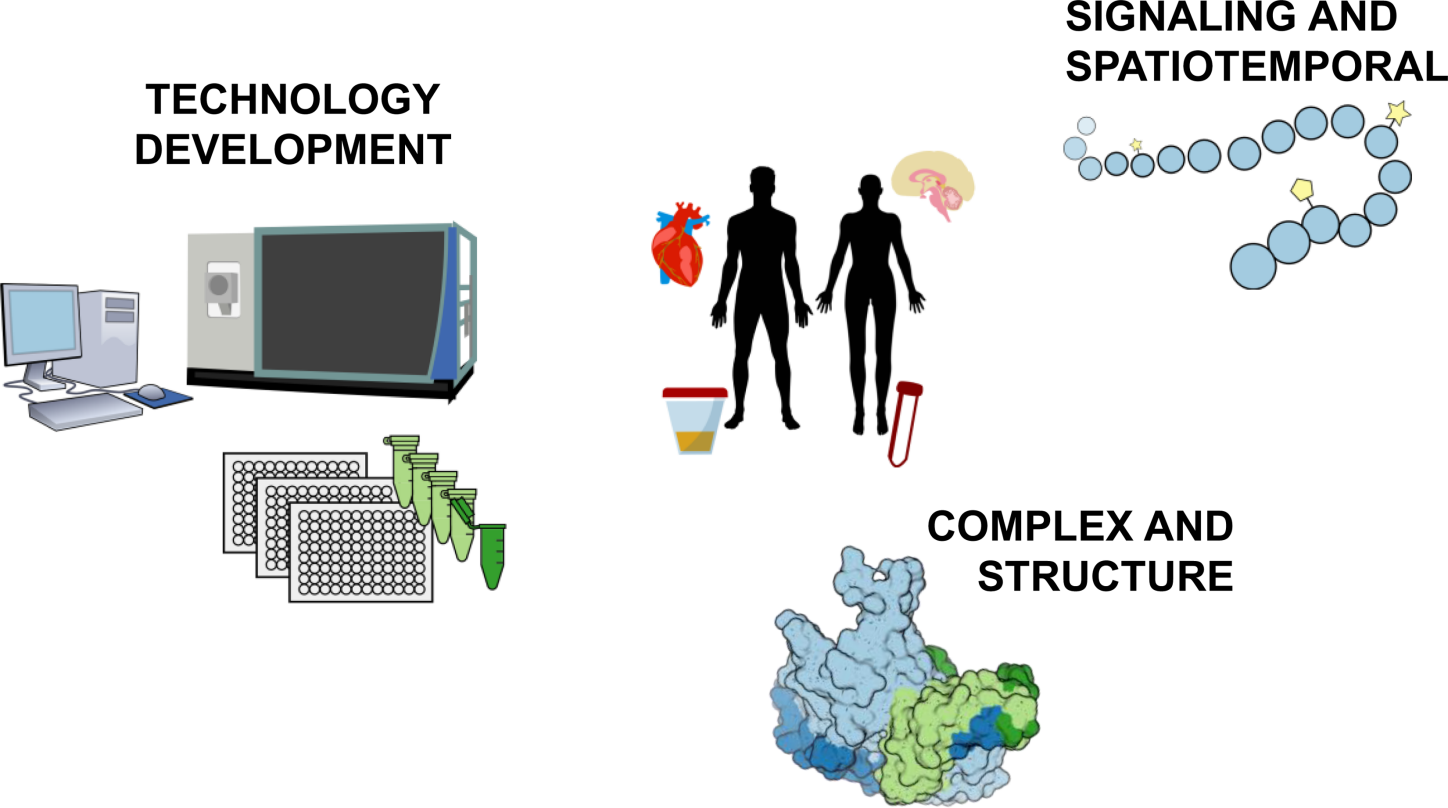
Biomedical research applications supported by recent MS-based proteomics technological advances. (clockwise from left) Technological advances in data acquisition and throughput have improved the ability to profile the proteomes of biological samples deeply and accurately, including clinical applications. New approaches for signaling and spaciotemporal protein dynamics improve the detection and quantification of post-translational modifications, proteostasis, and even enable single-cell proteomics. Finally, methods for studying protein complexes and structure facilitate studies of protein–protein and protein–drug interactions.

In this review, we examine the latest advances in the field so that biomedical researchers can quickly familiarize themselves with the newest proteomics workflows (Figure 2) that may be best suited for a given biological project.

## Technological developments in mass spectrometry

### New data acquisition innovation

Data-independent acquisitions (DIA) [19] or SWATH [20,21] is a systematic MS acquisition method that uses wide, nonspecific precursor isolation windows to activate all ions for collision in a given *m/z* range. Detailed reviews of DIA methodology can be found elsewhere [22,23], including peptide-centric approaches to DIA [24].

One advantage of DIA/SWATH MS over other methods is the resulting comprehensive and reproducible quantification of proteomes, making DIA/SWATH an attractive choice for broad quantitative studies of basic disease biology [22–25]. Machine learning models have alleviated earlier data analysis reliance on prior knowledge, specifically spectral libraries, by predicting MS/MS fragmentation spectra that allow researchers to build spectral libraries *in silico* [26,27] and the ‘chromatogram library’ approach for refining those predicted spectral libraries [28]. While there are several popular software options for analyzing DIA data [28–32], benchmark comparisons have thus far found the results comparable [33], freeing researchers to use whichever software they find most convenient. DIA/SWATH applications have been applied broadly wherever high-dimensional proteome quantification is interesting, including cancer biology [34], trisomy 21 Down Syndrome [35], differentiation [36], and stem cell biology [37].

Recently, an additional gas-phase separation methodology ‘trapped ion mobility spectrometry’ (tims) was combined with a fast scanning QTOF mass spectrometer, and the timsTOF PRO platform allows for novel acquisition methods called ‘parallel accumulation–serial fragmentation’ or PASEF [38] where the release of precursor ions can be synchronized with the quadrupole selection for fragmentation. Combining PASEF with DIA, diaPASEF [39] subsequently reduces complexity and depth of coverage as well as maintaining quantitative accuracy. Unlike typical DIA approaches that are based predominantly on chromatographic separation, diaPASEF suggests extending the comprehensive, systematic sampling of precursor ions by additional and orthogonal peptide separation applying ion mobility.

### Advanced isotope labeling strategies

**Figure 3.**
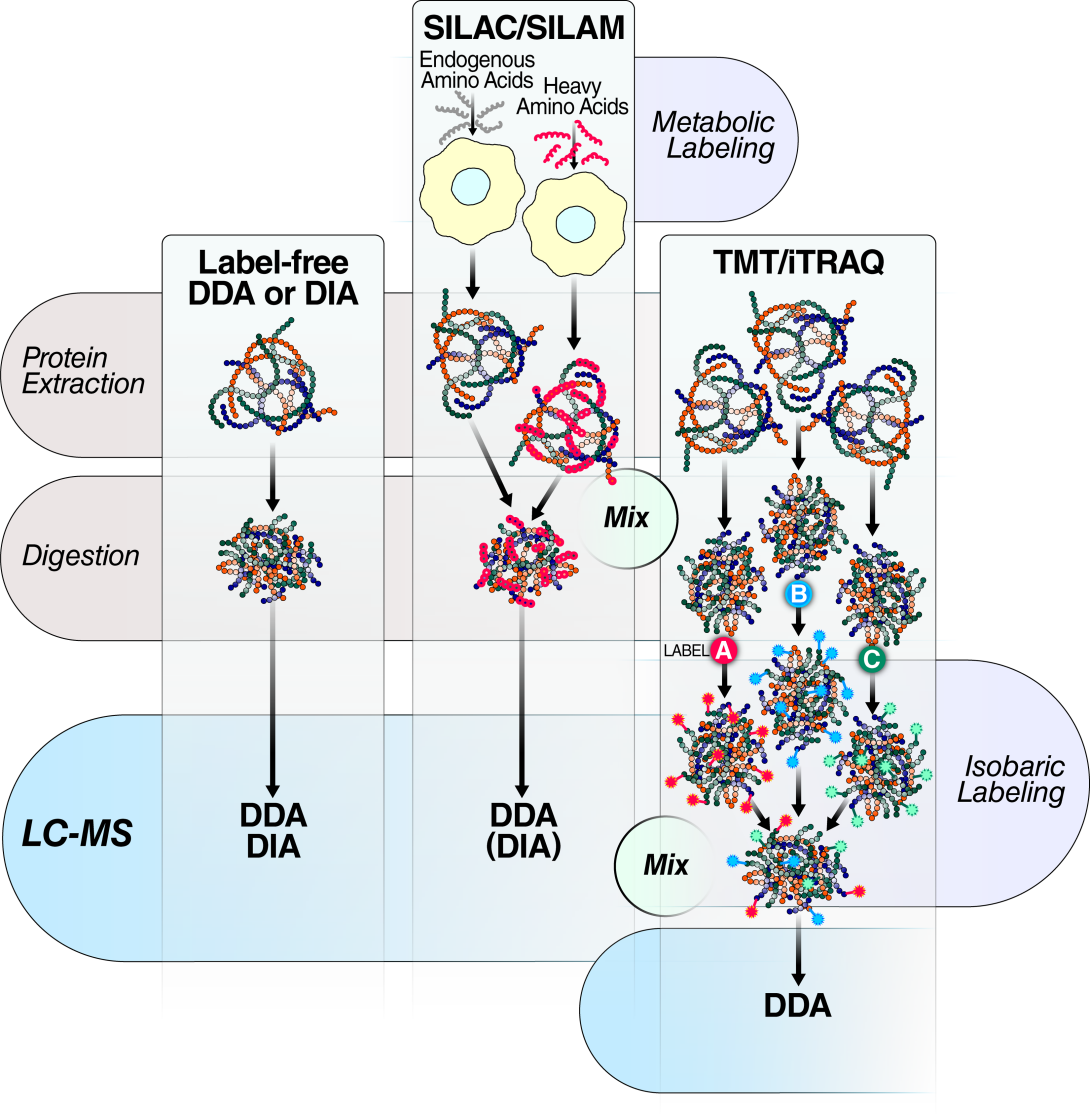
Overview of various comprehensive quantitative proteomics workflows. The shared workflow for most quantitative proteomics experiments typically involve protein extraction, proteolytic digestion, and finally analysis by LC–MS using either DDA or DIA acquisitions. The most general workflow, *label-free DDA or DIA*, requires no additional sample processing (*left*). *Metabolic labeling strategies* like SILAC/SILAM require that the heavy isotope label is incorporated into the metabolically active cells or organisms. After harvesting and lysis cells different samples are mixed and then digested and processed together prior to DDA or in some emerging cases DIA analysis (*middle*)*. Isobaric labeling strategies*, such as TMT and iTRAQ, require each sample in the multiplex to be individually digested and subsequently be reacted with the specific chemical label, prior to mixing all samples and DDA analysis *(right)*.

In addition to the label-free workflows described above, stable isotope labeling techniques have also improved, specifically allowing for increased multiplexing. Metabolic labeling such as stable isotope labeling with amino acids in cell culture/mammals (SILAC/SILAM) [5,40] and isobaric tagging methods such as TMT [6,7] and iTRAQ [8] are commonly used workflows. Label-based methodologies are useful as a multiplexing technique as shown in Figure 3. It allows the pooling of individually tagged/labeled samples to provide quantitative information by using isotope mass shifts or reporter ion signals that reflect the relative abundance of the original individual samples [41]. This process mitigates nonspecific and acquisition-specific artifacts that may occur with multiple acquisitions. SILAC/SILAM methods use metabolic labeling (typically heavy lysine and/or heavy arginine residues), where isotopes are incorporated into proteins *in vivo*, then protein lysates are mixed at equimolar ratios prior to downstream sample processing. Thus any downstream processing variability does not affect the ratios between the investigated conditions/samples. For isobaric labeling approaches typically 10–11 samples are lysed and digested individually, subsequently reacted with the labeling reagents (e.g. TMT/iTRAQ), then mixed at equimolar ratios (Figure 3).

With the latest advances for TMT, researchers can now even label up to 16 distinct samples with different TMT tags [42], pool them, and subsequently analyze them all in a single acquisition. Multiplexing can be increased to even higher levels [43] in anticipation of newer reagents in the future. Isobaric labeling strategies TMT workflows and the various improved generations of reagents have greatly improved MS multiplexing capabilities for relative quantification of different experimental conditions [44]. Proteomic depth of coverage is often achieved by off-line separation before MS analysis, at the cost of slower throughput.

However, one challenge to using TMT has been recognized and is referred to as ratio compression. To overcome these problems, additional elegant MS methodologies and scan sequences were developed. For example, interference in the TMT reporter ion region can be reduced by TMT-MS3 workflows in which TMT-labeled MS2 fragment ions are selected for further fragmentation in the ion trap (MS3) [45]. This workflow was further improved by a MultiNotch MS3 method that uses isolation waveforms with multiple frequency notches (i.e. synchronous precursor selection, SPS) to co-isolate and co-fragment multiple MS2 fragment ions, thereby increasing the number and intensity of reporter ions in the MS3 spectrum [46]. The TMT-SPS-MS3 workflow has gained interest, and several high-impact biological studies recently used this technology [47–50]. The throughput and depth in proteome coverage were showcased in a recent murine tissue-based study quantifying over 11 000 proteins [49]. TMT-SPS-MS3 analysis workflows have also been applied to large-scale phosphoproteomics projects (also called SL-TMT for streamlined TMT) [49], and a recent large analysis of mouse proteome tissue specificity [51]. Notably, modifications to isobaric labeling improved its efficiency so that it requires significantly less reagent. The significant reduction in the cost per acquisition make it more likely that TMT will be adopted in clinical tests or patient-specific oncological mapping [52].

### Improvements in sample separations and throughput

MS proteomics is usually paired with chromatographic separation, which reduces sample complexity and therefore improves the depth of proteome coverage. Liquid chromatography (LC) can be performed ‘off-line’ prior to analysis by mass spectrometry or ‘on-line’ because it is relatively straightforward to connect a LC to a mass spectrometer and ionize the eluting analytes in the ion source via electrospray ionization (ESI). Off-line fractionation is commonly performed for peptides, especially for multiplexed isotopically labeled samples (discussed in more detail above), using basic reverse phase (BRP; also called high pH reverse phase, HPRP) fractionation to separate peptides by hydrophobicity prior to analysis by on-line LC and MS [53]. While on-line LC at nanoliter flow rates (nanoLC) is commonly used because it requires less input material and may be more sensitive than higher flow rates (e.g. microflow LC), nanoflow is often considered less robust and potentially less reproducible. Modern LC systems combine the sensitivity of nanoLC with the robustness of microLC [54]. In the search for new biomarkers, the scale of proteomics experiments has grown exponentially and the robustness of microLC has been increasingly applied to improve throughput to thousands of biomedical samples [55,56].

While high-performance LC (HPLC) has been the separation method of choice for MS workflows, other separations developments are also improving the depth and breadth of peptide and protein identification from complex biosamples. A new data acquisition technique, BoxCar [57], improves precursor ion dynamic range and signal-to-noise ratios by first profiling the peptide precursor ions in a sample and then filtering the ions into segments of *m/z* ranges (‘boxes’) so that each MS1 segment shows an equal representation of ions across the full precursor range. With this methodology, Meier et al. [57] detected more than 10 000 proteins in only 100 min with sensitivity into the low-attomolar range. While BoxCar approaches were originally developed to augment DDA scan types, the principle can also be applied to DIA [58].

High-field asymmetric waveform ion mobility spectrometry (FAIMS; also called differential ion mobility spectrometry, DMS) can replace LC prior to introduction to the MS. FAIMS/DMS often separates analyte peptides or PTM isoforms that otherwise would co-elute and improves MS quantification by decreasing interference. FAIMS has been used to extend the dynamic range and accuracy of TMT-labeling quantification [59] and to reduce the length of LC gradients without sacrificing proteome coverage [60]. Another type of ion mobility mentioned above in the context of DIA, trapped ion mobility spectrometry (TIMS), provides capabilities to trap and release ions in a mobility-selective manner, thus categorized as a ‘Confinement and Selective Release’ methodology, while FAIMS and DMS approaches are considered and categorized as ‘Spatially-Dispersive’ ion mobility separation techniques. Briefly, a parallelizable 4D feature detection algorithm extracts peaks efficiently, and a new algorithm implemented in the free-to-use software MaxQuant called matching between runs (MBR) utilizes collisional cross section (CCS) values of MS1 features which significantly gains specificity from the extra separation dimension [61].

## Advances in signaling and spatiotemporal proteomics

### Post-Translational modifications

Post-translational modifications (PTMs), such as acetylation and phosphorylation, are widely used to regulate biological processes as recently comprehensively reviewed by Doll and Burlingame [62]. However, PTM analysis is challenging because the endogenous abundance PTM-containing peptides is typically much lower than that of unmodified peptides. Therefore, the modified peptides must be enriched from cells or tissues before MS analysis. Many PTM enrichment strategies involve antibody-based affinity purification (discussed below), which typically requires large amounts of starting material. Isobaric chemical tags, particularly the TMT method discussed above, are highly sensitive. They require less input material than conventional approaches for PTM analysis and have become a popular choice for studying modifications [7]. However, there are challenges. For example, ubiquitin remnants are chemically altered by the reagents, and thus, combining these two approaches for these types of studies has been difficult. One solution is the ubiquitylation enrichment protocol UbiFast. It labels the ubiquitin remnants (K-ɛ-GG) while they are bound to the anti-ubiquitin antibody and not exposed to the solvent. This method requires half the starting material and quantifies thousands more ubiquitylation sites than conventional approaches [63].

In addition, computationally detecting PTMs in mass spectrometry data is challenging. To solve this problem, researchers often perform an initial analysis with synthetic peptides. For example, the ProteomeTools project [64] aims to systematically characterize the human proteome by synthesizing over a million peptides, with initial data for 5000 peptides carrying 21 different modifications. Similarly, methods utilizing prior knowledge of the glycan structures released from glycoproteins are supporting glycomics studies [65]. A new informatic approaches called ‘open modification searching’ (OMS) focus not on specific predefined PTMs, but rather it detects shifts in peptide and fragment ion masses, allowing for the discovery of any possible PTM present in the data [66–68]. Although a powerful approach, OMS analyses often remain difficult to interpret biologically.

As these advances make PTM-centric experiments easier, efforts to describe the biomedical causes and consequences of protein modifications are increasing. For example, recent applications of PTM-centric mass spectrometry studies include the acetylome effects of deacetylase SIRT5, implicated in maintaining mitochondrial function during acute kidney injury [69]; malonylation and crotonylation have functions in inflammatory signaling [70] and regulation of chromatin remodeling [52], respectively.

### Approaches for studying proteostasis by protein turnover

Healthy proteostasis requires that proteins are synthesized and degraded at appropriate rates [71]. Newly synthesized proteins are measured by incorporating uncommon but metabolically viable amino acids, allowing researchers to elucidate how often specific proteins are synthesized and degraded. The most conventional experimental design for cell culture, commonly called pulse SILAC (pSILAC), uses media containing stable isotope-labeled amino acids, such as heavy arginine and heavy lysine. Interest in protein turnover for more complex model systems has reintroduced stable isotope labeling of amino acids in mammals [72]. Commercially available mouse chow containing the isotopes has been used with a focus on proteostasis in the brain [73,74].

In addition to pSILAC, non-canonical amino acid incorporation has emerged as a tool to elucidate tissue-specific protein turnover by coupling a mutant tRNA synthetase engineered to incorporate a specific non-canonical amino acid with a cell-type-specific driver [75]. Notably, when designing experiments with non-canonical amino acids, it is important to determine if the introduction of those amino acids causes unintended changes to the model system being studied [76].

### Single-cell proteomics

The amount of input material required for MS-based proteomics has made it unsurprisingly challenging to perform single-cell proteomics with MS. Perhaps the most straightforward single-cell approach to conventional bottom-up MS proteomics is to simply limit sample exposure to plastics. Coupling microfluidic chips to MS is not a recent innovation; in fact, the first microfluidics approaches for MS were introduced over two decades ago [77,78]. The rise of droplet-based microfluidics has emerged as a powerful tool in single-cell proteomics. Among the first nanodroplet-based devices for MS proteomics, nanodroplet processing in one pot for trace samples (nanoPOTS) measured over 3000 proteins from 10 cells using specially fabricated devices but conventional reagents and data analysis software [79]. Despite the impressive detection and quantification metrics reported by nanoPOTS, the lack of a commercially available nanoPOTS chip has reduced the adoption of the technique. nanoPOTS was followed up with an alternative approach using commercially available consumables, termed microPOTS [80], which can profile proteomes from as few as 100 cells but does not reach the same sensitivity as the nanoPOTS protocol. In contrast, another single-cell proteomics approach, Single Cell ProtEomics by Mass Spectrometry (ScOPE-MS) [81], employs TMT labeling to achieve single-cell sensitivity. ScOPE-MS creatively addresses two challenges. It reduces input loss by mixing the single-cell peptides of interest with so-called ‘carrier peptides’, and it uses acquisition methods that maximize ion transmission to reduce the limitations of instrument sampling [82]. Yet another single-cell method combines these two approaches: nanoPOTs droplet-based sample preparation and ScOPE-MS's isobaric tagging signal boosting [83].

There has not yet been a study to benchmark the detection sensitivity, precision, or quantitative accuracy of the various single-cell proteomics techniques, and so it is difficult to draw comparisons. As of this review, despite the functional importance of proteins, no single-cell proteomics technique is yet able to match the reproducibility or scale of sequencing-based analyses, such as sc-RNA-Seq, and adoptions of these approaches have been limited in part due to the high specialization and cost associated with droplet-based sample preparation and TMT tagging. However, beyond single-cell proteomics, methods to restrict MS analysis to specific parts of a cell or tissue are incredibly valuable and provide more accurate descriptions of cell function [84]. Subsampling techniques, such as fluorescence-activated cell sorting (FACS) [85,86] or laser-capture microdissection [87], are used to decrease the total input amount while simultaneously selecting for homogeneous cell populations. For example, the combination of precise dissections, FACS, and proteomic analysis has been used to garner the most accurate and delineated proteomes in the brain [88].

### Imaging mass spectrometry

Imaging mass spectrometry (IMS) is a robust label-free technique that enables users to visualize and characterize a broad spectrum of biomolecules, ranging from metabolites to complex peptides and protein formations and based on their spatial molecular distribution. Unlike traditional imaging methods, it allows the study of complex peptides and proteins without the need for existing knowledge of analytes [89–91]. The ionization technique for IMS is different than that of the ESI method described earlier. While multiple ionization technologies are compatible with IMS, matrix-assisted laser desorption/ionization-mass spectrometry is a popular choice. It allows multiplex analysis and can be used to image a variety of biomolecules in the same tissue section simultaneously [92]. Technological advances in IMS now allow comprehensive study of complex 3D cell-culture models, providing scientists the ability to effectively measure the efficacy of drugs before treatment and to observe the time-dependent penetration of chemotherapy drugs into tumor tissues [90]. An area of rapid development in IMS is data processing and statistical analysis [93–95], drawing newcomers to the IMS field by easing the burden of data analysis. Despite these computational advances, in-depth sample preparation protocols are needed for IMS to be utilized in more laboratories [91].

## Innovations in mass spectrometry-based protein complex and structure studies

Several general approaches detect and quantify protein–protein interactions, including size-exclusion chromatography (SEC), affinity purification, and proximity labeling (PL). A foundational review of these high-throughput technologies for protein interaction networks and others is found elsewhere [96]. Below, we focus on the most recent advances in these three approaches and highlight their differences.

### Size-exclusion chromatography

SEC itself was introduced half a century ago [97], but coupling SEC with MS for high-throughput analysis of protein complex components has only emerged in the past decade [98–100]. These approaches typically relied on label-based quantification strategies due to MS limitations; however, even more recently, advancements in computational proteomics have improved SEC-MS. One such workflow, hyperLOPIT, incorporates advances in labeling technologies discussed above to improve the spatial resolution of organelle proteomes [101]. Another computational analysis employs hierarchical clustering to group proteins with similar SEC elution profiles, so that identified proteins form the leaves of a dendrogram and protein complexes are determined by cutting the dendrogram at different levels [102]. A third method uses a semi-supervised support vector machine classification to determine protein complexes, taking as input co-fractionation data and additional information, such as published PPI datasets, co-evolution based on the assumption that true protein complexes will have conserved sequence and function, and other metrics that describe protein complexes [103]. A third example, CCprofiler [104], introduces a novel ‘complex centric’ analysis, which is the first to control error rates for protein complex analysis by using a statistical target-decoy method and also makes use of SWATH acquisition (discussed above) to improve the quantitative accuracy of SEC-MS.

### Affinity-purification mass spectrometry

**Figure 4.**
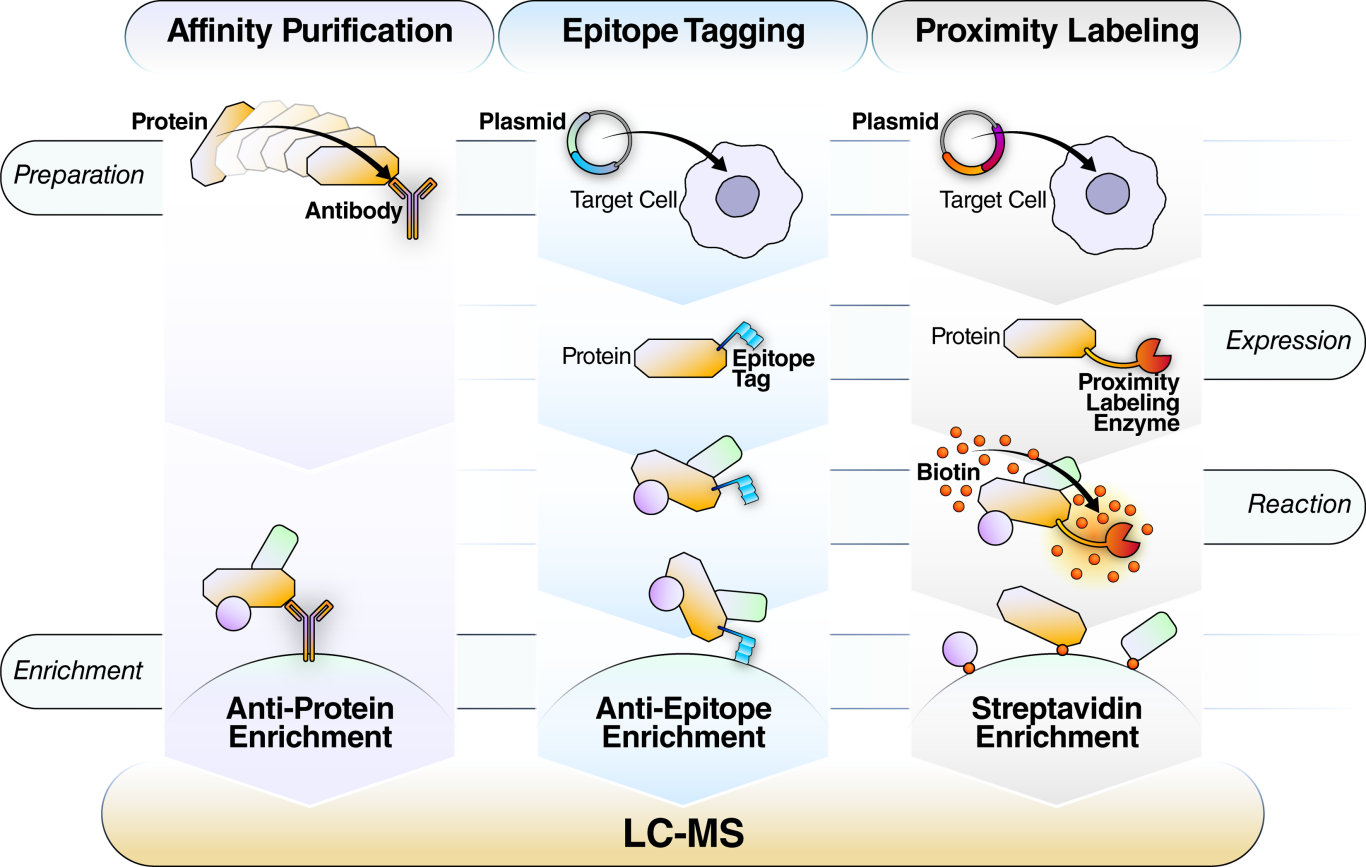
Strategies for assessing protein–protein interactions by mass spectrometry. The common goal for protein–protein interaction studies involves enriching and purifying a target protein of interest (the bait protein is indicated in yellow) together with its protein network/protein complex prior to identification by LC–MS. *Affinity Purification*: antibodies specific to the protein of interest typically are coupled to beads and are used to enrich the protein complex directly (*left*). *Epitope Tagging*: a plasmid construct with the gene of interest tagged with a common epitope is engineered, subsequently, the tagged protein is expressed in the model system, enabling the use of common affinity enrichment systems like streptavidin, anti-HA, or anti-FLAG (*middle*). *Proximity labeling*: a plasmid construct with the gene of interest fused to a proximity labeling enzyme such as APEX or BioID is engineered allowing for the covalent biotin labeling of any protein within a predetermined vicinity of the expressed fusion construct, followed by enrichment with streptavidin beads (*right*).

Affinity-purification mass spectrometry (AP-MS) is another well-established method for probing protein–protein interactions, in which antibodies raised against a protein of interest to co-immunopurify the protein and its interactors then analyze the enriched proteins by MS (Figure 4). Alternatively, epitope tagging can be used [105]. A monumental effort by the BioPlex project built tagged versions of, performed affinity purification for, and reported the PPI for nearly every protein in two cell lines [106]. Another application of AP-MS is in immunopeptidomics for novel vaccine development, where researchers seek to identify peptide antigens by purifying naturally processed peptide complexes with the major histocompatibility complex and analyzing the released peptide antigens by MS [107,108].

### Proximity labeling mass spectrometry

Finally, protein engineers have developed complementary means for localizing proteins and their interactors by fusing proteins of interest with PL enzymes (Figure 4). These enzymes chemically modify nearby proteins within a certain labeling radius with moieties, such as biotin, which is enriched for using streptavidin and analyzed by MS. The PL enzymes BioID [109], BioID2 [110], and APEX [111] have been successful in a variety of subcellular localization experiments, reviewed in detail elsewhere [112–114]. To address labeling efficiency limitations, recent work in protein engineering produced the more efficient PL enzymes TurboID and miniTurbo [115]. Adoption of PL into the community is supported by plasmid and lentiviral tool kits [116]. PL-MS studies primarily employ DDA techniques to identify proteins and typically use label-free spectral counting for protein quantification, although SILAC [117,118] and targeted approaches [119] have been used to improve quantification.

### Hydrogen-deuterium exchange mass spectrometry

In addition to PPI analyses, purified proteins obtained through any of the above techniques can themselves also be analyzed by MS to determine their combinatorial PTMs and their structures using hydrogen-deuterium exchange mass spectrometry (HDX-MS) [120]. In this highly technical procedure, the purified protein is exposed to deuterated water to exchange solvent-accessible hydrogens with deuterium, causing a mass-shift that is detected by MS. Developments in microfluidics [121] and nano-electrospray theta-capillary technologies [122] improved upon back-exchange issues. Although HDX-MS is relatively challenging, it has useful biomedical applications in pharmacological drug discovery and development to determine structure-activity relationships of small molecules and their protein targets and to classify ligands by their functional selectivity [123–125].

### Top-down mass spectrometry

Although the approaches described above all employ ‘bottom-up’ MS methods in which proteins are enzymatically digested prior to analysis, ‘top-down’ MS [126] is also used in protein interaction studies, especially in the context of determining proteoforms [127] and complex stoichiometry. Unlike the bottom-up approach that measures peptides not proteins, top-down mass spectrometry preserves all the structural information at the protein level, such as allelic variance, transcript splicing, and combinatorial PTMs. While top-down proteomics relies on the prefractionation of complex samples to purify the protein or complex of interest, new separations approaches, such as modified SEC purification techniques [128] and GELFrEE [129], preserve proteins in their native form. Native mass spectrometry, in contrast with most denaturing top-down proteomics approaches, is performed under native conditions, specifically at pH 7, to preserve protein complexes, capturing information about component stoichiometry and the spatial organization of those components [130]. Advances in instrumentation [131,132] and informatic analysis [133] eased the entry barrier for performing top-down and native proteomics.

## Conclusion

With modern advances in mass spectrometry and new instrumentation features, novel workflows now enable previously impossible biomedical applications. Even a few years ago, PL or single-cell proteomics seemed distant future but are now becoming much more streamlined. We also anticipate that many new technological innovations will be implemented in new generation mass spectrometers in the next coming years.

## Perspectives

- MS-based proteomics fills the need to reliably ascertain information about a sample; the desired information guides the selection of the methodology used. Now more than ever, MS methods have a range of capabilities broad enough to support just about any proteomics investigation.
- A common theme in the efforts to improve MS techniques is to combine two or three methods and optimize them so they can provide better results in tandem than either one can provide separately. Another theme is to push the limits of a single method in terms of its reagents, machines, and technique capabilities.
- Ultimately, in the future, we envision widespread implementation clinical MS and the development of personalized medicine employing these MS-based proteomics technologies.

## Abbreviations

DDA: data-dependent acquisition
DIA: data-independent acquisitions
DMS: differential ion mobility spectrometry
ESI: electrospray ionization
FACS: fluorescence-activated cell sorting
FAIMS: high-field asymmetric waveform ion mobility spectrometry
HDX-MS: hydrogen-deuterium exchange mass spectrometry
IMS: imaging mass spectrometry
LC: liquid chromatography
MS: mass spectrometry
nanoPOTS: nanodroplet processing in one pot for trace samples
PASEF: parallel accumulation–serial fragmentation
PL: proximity labeling
PRM: parallel reaction monitoring
pSILAC: pulse SILAC
PTMs: post-translational modifications
QTOF: quadrupole time of flight
ScOPE-MS: Single-cell ProtEomics by mass spectrometry
SEC: size-exclusion chromatography
SILAC: stable isotope labeling with amino acids in cell culture
SPS: synchronous precursor selection
SRM: selected reaction monitoring
TIMS: trapped ion mobility spectrometry
TMT: tandem mass tagging
